# Annotations

**Published:** 1905-11-25

**Authors:** 


### ANNOTATIONS.

### Death after Operation for Adenoids.

On Monday an inquest was held upon the body of a boy who had died as the result of an operation for adenoids, performed at the Manchester Eye and Ear Hospital. It appears that the patient was submitted to operation on November 14, and some three-quarters of an hour after the removal of the adenoids he was examined by the surgeon and permitted to return home. According to his parents, the bleeding, which naturally occurs as the result of the treatment, continued, and the patient gradually sank and died on the following day. It does not appear that there was in this instance any lack of ordinary care on the part of either the hospital or the surgeon. Nevertheless the case is well worthy of attention, for it provides an object lesson which should come home to many hospitals and many out-patient surgeons. So frequently is the operation for adenoids performed, that to admit the patients to the wards even for one night would seriously impair the capacity of the hospitals for dealing with more serious cases. But because it has been found expedient to perform the operation in the out-patient department, it does not follow that ordinary precautions can be dispensed with. On the other hand, the recognised disadvantage of such out-patient operations should lead to an exercise of extreme caution both in recommending and in carrying out such operative treatment. We are afraid that a deplorable custom of recommending the removal of adenoids as soon as there is any trace of overgrowth of these structures, is becoming established. Such hasty, not to say frivolous, surgery is to be condemned most strongly. The present attitude of over-weening confidence in the success of surgical methods is constantly courting disaster. It is only by giving prominence to such an example as the one which the Salford coroner has been called upon to investigate, that a proper sense of proportion is likely to be secured.

### The "Adopted" Child.

The Home Secretary is to be asked to receive a deputation from the Association of Poor-law Unions on the subject of infant life protection. As the law at present stands, inspection is made only of homes where two or more children are kept at nurse. The great majority of nurse children are illegitimate, and in many cases their mothers would be only too thankful to be relieved of a burden which handicaps them sorely in the struggle for life. Women in such a case do not always inquire very closely into the credit of the person who undertakes the care of their child. But the foster-mother who takes only one child in to care for at present escapes the eye of the law, and though she may get into trouble if a child's death can be traced to her neglect, there are many ways in which a child may be weakened until it dies, without a case being proved against the person who has charge of it. The administration of ill-chosen food will always pass for ignorance, where absolute starvation might be risky. Special doubt exists as to the *bona fides* of those people who offer to "adopt" a child if a small premium be paid. The few pounds demanded will never maintain a child for so long that the receipt of it would make any difference to a person who really wanted a child to bring up as her own, and, as a rule, the advertisement of these would-be foster-mothers adds to the words "small premium," these others "no after claim." Which means that in many cases the child will be shrewdly done to death before the amount of the premium is spent. The careless or criminal baby-farmer will not be effectively controlled until there is registration and inspection for every place where a child is taken in and kept for payment. It is also desired that the age at which inspection ceases should be extended to seven years. At present, inspection ceases when the child is five years old. At that age it should come under the purview of the school-attendance officer, but the new regulation which delays the admission of a child to school under the age of five years—an admirable regulation in itself, both as regards the health of the children and the economy of public money—may in the case of nurse children just leave an interregnum which may be fatal to life. There is little doubt that when public opinion is aroused on the matter the necessary extension of power will be given, but it will be to the credit of the law if it does not wait for public opinion to press it on.

### The Encouragement of Small-pox.

On Monday afternoon thirty-three men who have persistently refused to obey the law in respect to vaccination were, after a long period of immunity, incarcerated in Derby prison. The number of defaulters who declined to pay the penalties incurred by disobedience to the Act was originally thirty-five, but at the last moment two discharged the claim because, as it is stated, their employers told them that if they went to gaol they would lose their work. We do not know if the majority ran that risk, but men who persist in disobeying a law which provides absolute security against a terrible disease, are not entitled to much consideration from their masters. We note that the prisoners are served with a good meal each day by the Anti-Vaccination League, who have also taken upon themselves the responsibility of looking after the wives and families of the prisoners. This is fitly supplemented by the misplaced leniency of the local magistrates, who, while committing the men for seven days, have decided to release them on Saturday, two days before the expiration of their term. Worst of all, it seems that but for the insistence of the Home Office the warrants issued against the defaulters would not have been served. It was not until an intimation was received from London that the police grant would be withheld if the warrants were not executed, that the goods of the culprits were seized, and then the sale that followed was of a farcical character. We are afraid that nothing short of an epidemic of small-pox will suffice to make the Derby people realise the importance of treating anti-vaccinators as a distinct menace to the public health.

